# Atmospheric Pressure in the Retention of Entire Dentures

**Published:** 1885-07

**Authors:** W. B. Ames

**Affiliations:** Chicago, Ill.


### ATMOSPHERIC PRESSURE IN THE RETENTION OF ENTIRE DENTURES.

BY W. B. AMES, D. D. S., CHICAGO, ILL.

The principle of atmospheric pressure is definite enough, and easy of understanding and of application to solids of definite form, or to liquids, but when applied to bodies of variable form and resistance much attention to detail is called for. As in any branch of our art, it is not the adherence to general principles as much as the attention to detail and conditions that brings success.

In looking over the literature at command, I find that very little has been written on the subject of late years, and what has been written at any time has been of an indefinite nature. The instruction in the various text books is extremely vague as to the application of the principle of atmospheric pressure to the retention of artificial dentures. They speak of its being applied in cavity plates and contact plates, saying nothing about the conditions on which the success of each method depends. For instance, Richardson says, "where practicable, it is better to make a central chamber, the size and location of which will depend upon the condition of the soft parts," without specifying the condition.

Much has been said of the advantages and injurious effects of this central chamber, and its usefulness and uselessness much discussed. About the year 1862, a sort of controversy on this subject was indulged in by some of the lights of the profession. Dr. John Allen published a thoroughly scientific article, in which he claimed that the only office of the central chamber is to prevent the rocking of the plate upon the hard parts in the centre of the palate. Dr. Pease, in a very extensive discussion, urges the necessity of the cavity over the centre of the palate, the amount of pressure derived being proportional to the size of this cavity. Dr. Ambler Tees, in a paper read before the Illinois State Society, makes a statement to the effect that he has in several instances swaged two plates for the same mouth, one with a chamber and one without, and found the pressure so much stronger in the former that in every case he discarded the latter. Now in this method of Dr. Tees, we have no statement to show that he made his plain plate of different size, shape, or relations from his cavity plate. Other cases have been cited where mouths have been satisfactorily fitted with *plain* plates, after various failures with plates depending upon the suction of a central cavity had been met with, but the citer never describes the relations of his plate to the tissues beneath and about it, which would indicate that a stroke of luck was to be credited with the results. In these cases I hold that something more than the adhesion of contact or capillary attraction must have been secured, either rationally or otherwise, and that this *something* is a powerful pressure of the atmosphere. This pressure I am able to obtain with plates devoid of any central cavity or air chamber, by a method which I have employed for sufficient time to be satisfied that it is applicable to any case where there is firm tissue over any part of the palate or alveolar ridge.

Various methods of trimming the impression and model to relieve and equalize pressure have been given from time to time, the best of which I consider to be that of Dr. Holbrook, of Kenosha, Wisconsin, which method he patented, and divulged to the profession for value received only, and under promise of secrecy. This method, as exposed by the inventor, seemed most applicable to lower dentures, his instructions for the treatment of upper cases not being sufficient to cover all requirements for best results in the majority of cases.

His main points were to trim the impression where the jaw is hard, and the model where the jaw is soft, over such parts as are to be covered by the plate. Thus, in most cases, he would pare away the *impression* to correspond with the firm surface at the central region of the palate, and cut a groove across the *model*, anterior to the line of attachment of the soft palate. This groove, which would mark the posterior termination of the plate, would be deepest at each side, and shallow directly over the median line. This is the substance of his instruction, the aim being to avoid rocking and to obtain perfect contact at the posterior edge.

In most cases the variable resistance of the tissues anterior to the soft palate is the condition that is fatal to best results by this method. In a few cases the tissues are sufficiently lax over the posterior portion of the hard palate for this groove to be made of the same depth entirely across, but generally the laxity begins at the margin of the bone, especially at the median line, so that in most cases to have the edge of the plate displace the soft tissues unifoımly, it is necessary to extend the plate a little posterior to this margin.

My course of procedure for upper dentures is to obtain a good impression of the jaw with any material whatever, being especially careful to have the impression extend a little beyond the margin of the hard palate, well up beneath the lip and cheeks and around the maxillary tuberosities. The material for the impression should be of such a consistency as will compress all soft or lax parts, or the model obtained from it should be pared down at the location of these parts. Then should be ascertained and marked out upon the model the exact location of the points where the laxity of the soft tissue of the palate commences. Posterior to this line an obtuse groove should be made across the model, extending from the region behind one tuberosity to that opposite. This groove should terminate at each side, at a point where the edge of the plate will be well covered by the buccal tissues, thus leaving no possibility of the entrance of air beneath the plate. The depth of the groove must depend upon the laxity of the parts. It should be of the depth to which it is desired the plate shall displace the tissues.

By constructing a plate over this model, allowing it to extend posteriorly into the groove and well up beneath the lip and cheeks, it will not be apt to exert any perceptible influence on the surface of the mouth when there is no force applied to displace it, but immediately upon its displacement from contact with any portion of the surface a vacuum is produced, from the fact that all edges of the plate were slightly displacing lax tissues that will follow the edge downward, and atmospheric pressure be obtained over all firm parts of the jaw. When removal is desired the force can be broken by raising the lip, thus admitting air beneath the plate.

The ideal atmospheric pressure plate is one which offers the maximum resistance to displacement with the minimum of bad results from traction upon the tissues. By the above method a plain plate can be made to embody in a greater degree all of the advantages of a cavity plate, without the bad effects of the cavity.

A few patients cannot tolerate a plate extending upon or near the soft palate, on account of the nausea produced. In such cases the groove can be formed as far forward as is necessary, and the posterior edge of the plate made to consist of soft rubber, which from its flexibility will give an accommodating contact, to answer the same purpose as the yielding soft parts with the other form of plate.

This principle might be applied to an occasional lower denture, where the ridge is broad and firm, but such cases are rare.

Plates of any material can be made after this method. It is especially suited for continuous gum work, and for other metallic plates when made with rubber or celluloid attachment, all edges being made thick and rounded to prevent irritation of the soft tissues. It is also consistent to retain healthy roots in the jaw, as they do not interfere, and if situated at the four angles will improve the masticating capacity of the apparatus.

